# Baleen Hydrodynamics and Morphology of Cross-Flow Filtration in Balaenid Whale Suspension Feeding

**DOI:** 10.1371/journal.pone.0150106

**Published:** 2016-02-26

**Authors:** Alexander J. Werth, Jean Potvin

**Affiliations:** 1 Department of Biology, Hampden-Sydney College, Hampden-Sydney, Virginia, 23943, United States of America; 2 Department of Physics, Saint Louis University, St. Louis, Missouri, 63103, United States of America; UC Santa Cruz Department of Ecology and Evolutionary Biology, UNITED STATES

## Abstract

The traditional view of mysticete feeding involves static baleen directly sieving particles from seawater using a simple, dead-end flow-through filtration mechanism. Flow tank experiments on bowhead (*Balaena mysticetus*) baleen indicate the long-standing model of dead-end filtration, at least in balaenid (bowhead and right) whales, is not merely simplistic but wrong. To recreate continuous intraoral flow, sections of baleen were tested in a flume through which water and buoyant particles circulated with variable flow velocity. Kinematic sequences were analyzed to investigate movement and capture of particles by baleen plates and fringes. Results indicate that very few particles flow directly through the baleen rack; instead much water flows anteroposteriorly along the interior (lingual) side of the rack, allowing items to be carried posteriorly and accumulate at the posterior of the mouth where they might readily be swallowed. Since water flows mainly parallel to rather than directly through the filter, the cross-flow mechanism significantly reduces entrapment and tangling of minute items in baleen fringes, obviating the need to clean the filter. The absence of copepods or other prey found trapped in the baleen of necropsied right and bowhead whales supports this hypothesis. Reduced through-baleen flow was observed with and without boundaries modeling the tongue and lips, indicating that baleen itself is the main if not sole agent of crossflow. Preliminary investigation of baleen from balaenopterid whales that use intermittent filter feeding suggests that although the biomechanics and hydrodynamics of oral flow differ, cross-flow filtration may occur to some degree in all mysticetes.

## Introduction

As early as 350 BC, Aristotle differentiated mysticetes from odontocetes by their oral filter. In *De Partibus Animalium* (On the Parts of Animals) [1] he described mysticetes as having bristles or setae in place of teeth; in *Historia Animalium* (History of Animals) [2] he noted that these hairs resemble hog bristles. We can speculate that Aristotle understood the general function of this material as a filtration device. Nonetheless, we have yet to comprehend details of baleen operation. It remains a biomechanical “black box.” Entries in the *Encyclopedia of Marine Mammals*, the leading reference work, offer bland generalities on the topic of mysticete filtration: “Periodically the mouth is closed and plankton are removed from the baleen by the tongue, and ingested” [3]; “Baleen whales… force the water containing food out through the baleen plates, and then transfer trapped food back to the gullet. The tongue is presumed to be involved” [4]; “Water is expelled by the pouch and tongue through the still exposed baleen plates. Once the water is expelled the prey is swallowed” [5]; “The tongue and the elastic properties of the ventral walls of the throat act in concert to force water out through the baleen” [6]. Any shortcomings in our understanding of mysticete filtration are not the fault of the experts who wrote these entries; rather, they stem from limitations (logistical, legal, financial) constraining marine mammal research.

Advances in the study of mysticete feeding have been made with digital tags deployed on foraging whales [7–16], often combined with fluid dynamics modeling [17, 18]. Data from depth recorders, accelerometers, hydrophones, and video reveal links between mysticete locomotion and prey engulfment. However, these approaches have yet to resolve the dynamic forces and flows *within* the whale mouth or the direct action of the baleen filter.

Balaenid whales (bowhead, *Balaena mysticetus* Linnaeus 1758, and right, *Eubalaena* spp. Linnaeus 1758) feed via unidirectional water flow through a partially open mouth, enabling continuous ram filtration, in which baleen both captures and retains prey. This is most commonly observed at the surface but can occur at all levels of the water column. Because this process operates exactly like the skimming of a continuously propelled or towed net that removes items from water, the balaenid feeding mechanism is referred to as skim feeding. This is in contrast to intermittent filter-feeding mysticetes (rorqual and gray whales), in which baleen retains prey caught by the expanded buccal cavity during the engulfment of a large mass of water and prey in a single mouthful that is filtered only after mouth closure [19, 20].

**Fig 1:**
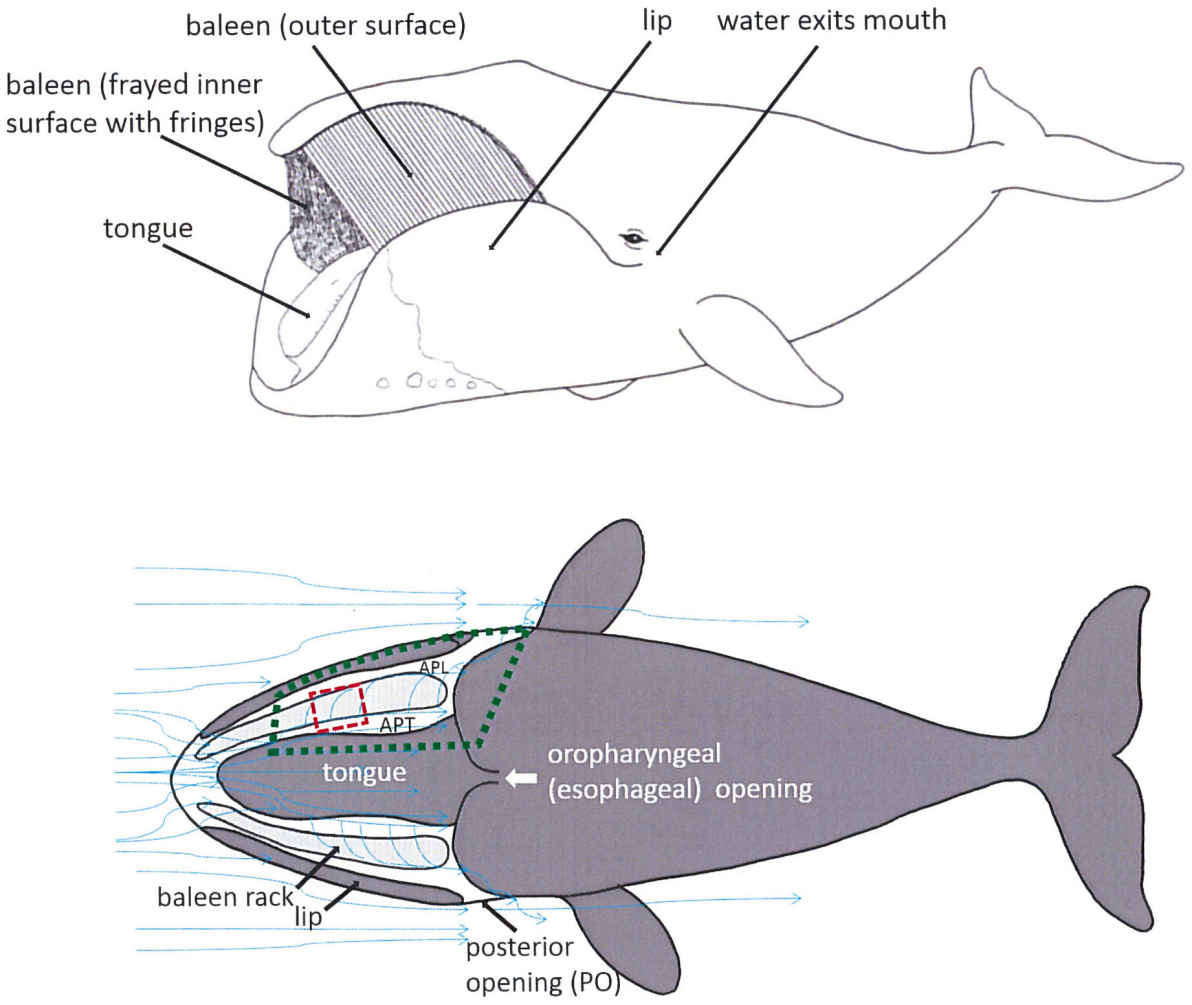
External and schematic (dorsal cutaway) views showing position of paired racks of 300 serial baleen plates between tongue and lips. In dorsal cutaway view with oral roof removed (bottom of figure), blue arrows indicate direction of water flow though and around baleen filtering apparatus in life as well as in experimental flow tank trials and computational modeling calculations (hypothetical but predicted from data of current study and previously published experiments [23, 25]). Water can flow anteroposteriorly (AP) within mouth along the tongue (APT channel) or the lip (APL channel). Dashed red box indicates location of shortened mini-rack used in “free flow” conditions (without tongue and lips); dotted green box shows more complex “bounded” conditions including tongue and lips. Filtered water exits the mouth via paired posterior openings (PO). Oropharyngeal opening which leads to esophagus lies near oral floor caudal to the tongue root.

Numerous features of balaenid oral morphology, unique among mysticetes, promote continuous, unidirectional flow of prey-laden water through the mouth, including the subrostral gap (a cleft between left and right baleen racks below the tip of the rostrum), orolabial sulcus (a gutter-like depression medial to the lip), and high semicircular lips extending above the mandibles to enfold the narrow, highly arched rostrum [20, 21]. The exceptionally long (up to 4 m), springy, finely fringed baleen is apparently cleaned of accumulated prey (by hydrodynamic flushing, mechanical scraping via lingual motion, or shaking of the head [22]) at varying intervals depending on prey density. Filtered water exits the mouth posteriorly through jetport-like openings at the angle of the gape, the so-called posterior opening (PO), just anterior to the eye [23] (Fig 1). Filtration is ram-powered by the whale’s forward motion, which by virtue of body taper (Fig 1) results in a zone of low pressure near the PO, thereby creating a decreasing internal pressure gradient [23]. From the low speeds employed, continuous skim feeding appears to be a low-drag, low-energy mode of foraging, far less demanding energetically for balaenids than the intermittent lunge-feeding of balaenopterid (rorqual) whales, which incur metabolic cost as high as 15 times their BMR at the largest body sizes, or 30 times that of terrestrial mammals of the same mass [18].

**Fig 2:**
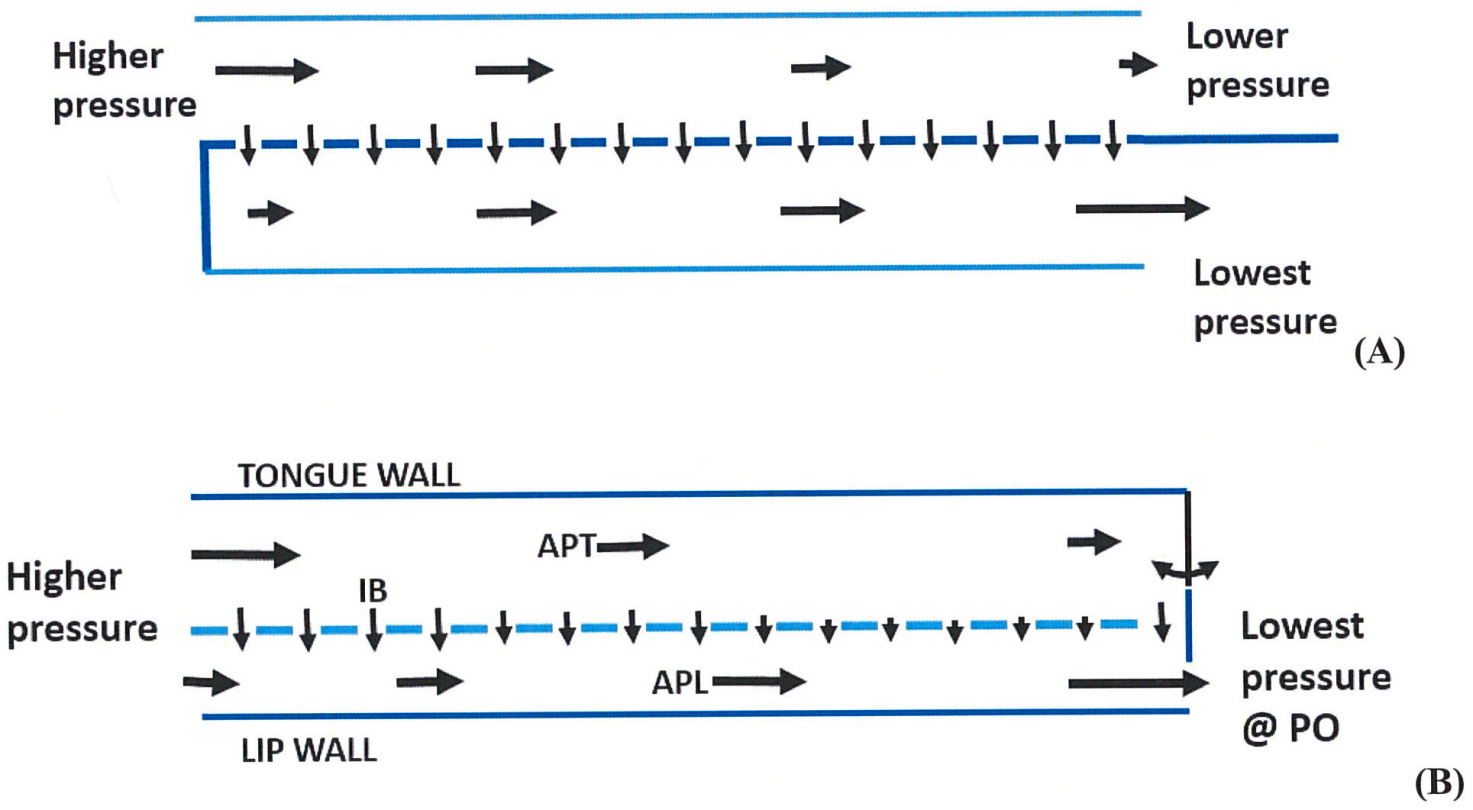
Schematic diagram comparing arrangement of cross flow filtration (CFF) apparatus in general (A, top) vs. CFF in balaenid whale mouth (B, bottom). Flow speeds and directions are represented by arrows, with the longest and shortest corresponding to the fastest and slowest speeds respectively. Dashed lines represent mesh screens used for industrial CFF (in A) or an array of ~300 fringed keratinous baleen plates suspended from the palate of balaenids (B). With the latter, anterioposterior flows on the lingual and labial sides of the baleen rack are shown as *APT* and *APL* respectively; lateral flows through baleen are shown as *IB* (intra-baleen). In both cases, and despite the drop of pressure longitudinally, the flows above the filter lose speed via mass loss through the lateral flows, per conservation of the mass rate (Fig 3); in contrast, longitudinal (AP) flows below the filter gain speed following its merging with IB flows. The double arrow on the right in (B) symbolizes the possibility of an open esophageal opening for engulfment of the filtered slurry.

Flow tank experiments [24, 25] indicate that intraoral flow patterns during continuous flow scenarios in balaenid whale mouths are more complex than had previously been assumed. Differential movement of suspended particles in three planes—anteroposterior (AP), dorsoventral (DV), and mediolateral (ML)—has been observed depending on the position within the baleen rack: specifically, whether the baleen in question is nearer to the mouth’s anterior opening, and thus the front of the flow field, or closer to the posterior oral orifice where filtered water exits. Such investigations [23–25] further suggest the existence of two longitudinal, anteroposterior flows, namely APT flow moving in the gap between the tongue and medial side of the baleen rack, and APL moving within the channel formed by the lips and lateral side of the baleen rack (Figs 1 and 2), with the APL receiving water flow from the APT route via mediolateral or intra-baleen (IB) flow.

In the standard mysticete model, baleen acts via “dead-end” or throughput filtration (TPF) by directly sieving prey items [26–28] as water flows laterally through baleen plates (laminae) and baleen fringes (also called bristles, hairs, fibers, and filaments [29]). A problem with this traditional view is that TPF involves a single, through-filter flow from inlet to outlet, a pattern that is clearly at odds with the morphology and hydraulics of the balaenid mouth (Fig 1), where flows on each side of the mouth run along and across a filter made by the serial array of ~300 baleen plates and mat of baleen fringes [25]. More likely than TPF is a cross-flow filtration (CFF) process (Fig 2), in which the bulk of prey-laden water is split into a particulate-carrying flow moving quickly and tangentially to the filtration surface, concomitant with a filtration process whereby a slower current, mostly free of prey items, flows directly through and past the filtration surface [30–32]. Hydrodynamically, CFF is enabled by twin pressure gradients established along and across the filtering surface (Fig 2). Although CFF involves some sieving, CFF gains substantial efficiency over TPF by using large filtering surfaces. This is not because CFF requires a larger filter relative to TPF; rather, less drag is generated with CFF relative to TPF because the filter is oriented parallel rather than perpendicular to the oncoming external flow, as well as to most of the internal flow. In other words, CFF generates less drag by virtue of filter orientation alone, and thus allows for the use of larger filtering surfaces. Moreover, and as shown here, this in turn generates considerably slower through-filter flows and substantially longer times until the filter becomes clogged. A larger filter also would allow more efficient capture of small prey aggregated in schools or patches. This is crucial in balaenids, where large, continuously filtering surfaces are necessary to optimize prey capture rates and volumes, with CFF allowing filter surfaces to be aligned not laterally but longitudinally within the body, thereby maintaining body streamlining.

**Fig 3:**
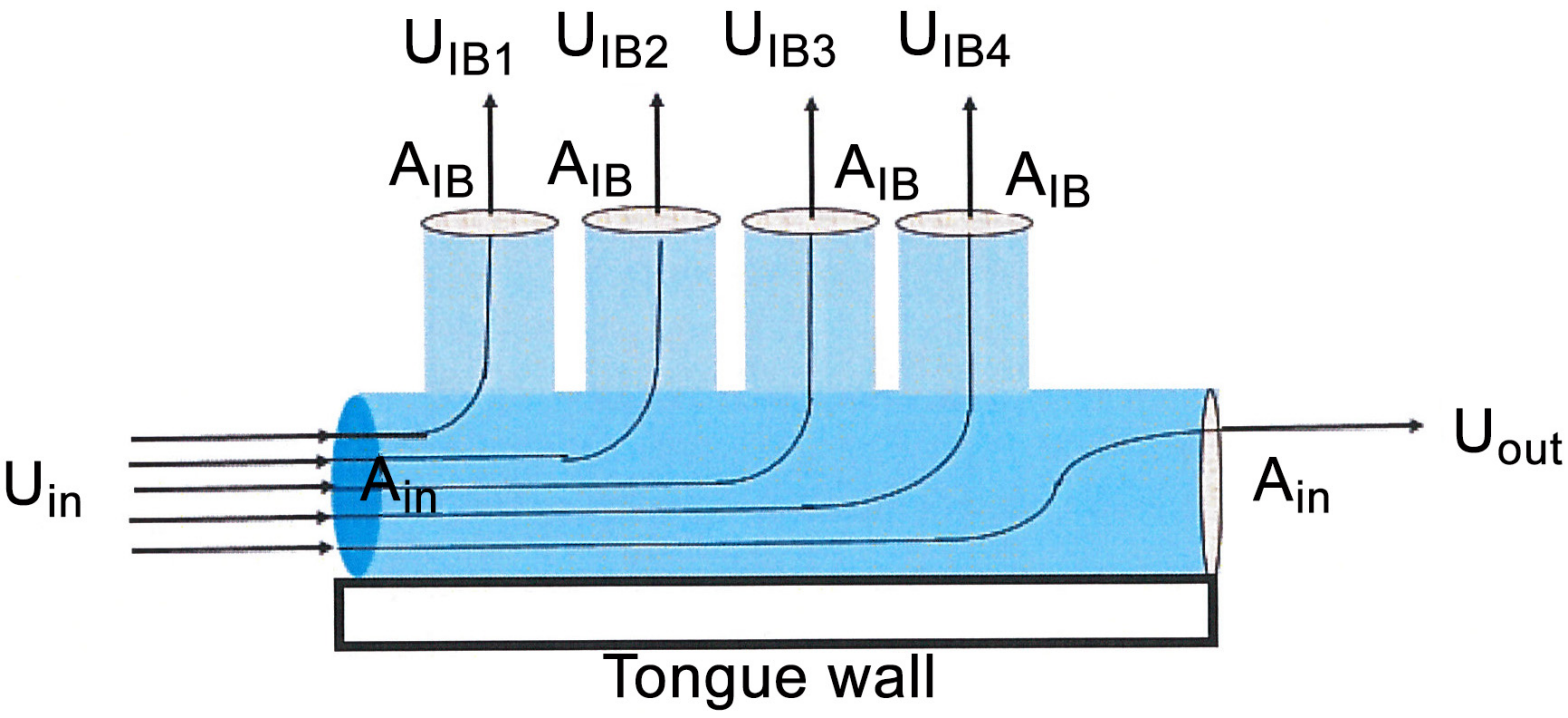
Schematic diagram of weakening axial flows (APT flows in balaenids) via mass loss via IB flows through a cross filter. The principle of mass flow rate conservation dictates that the exiting axial flow (*U*_*out*_) becomes weaker after sequentially losing mass to filter surface outlets (of cross-section area A_*IBi*_). In other words, and with the speeds through the outlets denoted as *U*_*IBi*_, the principle dermands that the inlet and outlet flow speeds be related as follows in the case of a schematic four-plate baleen system: *ρU*_*out*_*A*_*in*_ = *ρU*_*in*_*A*_*in*_ *- ρA*_*IB*_ *U*_*IB1*_ *- ρA*_*IB*_ *U*_*IB2*_ *- ρA*_*IB*_ *U*_*IB3*_ *- ρA*_*IB*_ *U*_*IB4*_*) = ρU*_*in*_*A*_*in*_ *- ρA*_*IB*_ *4‹U*_*IB*_*›*. Symbols *ρ* and *‹U*_*IB*_*›* correspond to the flow’s mass density and to the IB canal flow speed averaged over all four outlets, respectively.

CFF is widely used in industrial beverage filtration (Fig 2), where filtered liquid is the desired portion and retained particles are unwanted [32]. In mysticete oral filtration, in contrast, the concentrate (aggregated prey items) is the desired portion; their collection near the posterior end of the mouth by the opening of the oropharynx, where prey could be swallowed in a dense slurry, would be a major advantage (Figs 2 and 3). In CFF most particles do not directly contact the filter surface in the high numbers seen in TPF, leading to little direct sieving. Also in CFF, most particles flow in shear along the filter boundary at speeds significantly higher than the cross-filter surface flow [30–32]. This retards particle buildup against the filter’s pores and prevents filter clogging while enhancing concentration of filtered particles near the filter’s “output,” in this case near the oropharyngeal opening to the esophagus. Another potential benefit is the greatly reduced speed of prey-laden flow near the oropharynx, which would facilitate bulk swallowing of prey with a minimum of seawater. Thus balaenid CFF would enable simultaneous removal of retained particulate matter (prey) via swallowing concomitant with removal of liquid filtrate as outflow through the PO (Fig 1), a process that in TPF instead involves not concurrent but sequential steps.

Relative to TPF, CFF offers increased flow with decreased resistance, so that there would be less drag on the locomotion powering the ram filtration [23, 25]. Moreover, as explained here, the extreme body size and large filtration area of balaenids further enhances CFF by generating slower through-filter flows and longer clogging times. Thus, as increased filtering efficiency yields more prey, evolutionary pressure promotes larger body size.

Oral CFF has been documented in ram-feeding bony and cartilaginous fishes, where it concentrates food and prevents clogging of gill rakers [33–37], and has been cited previously as a possibility for mysticete filtration [8, 25, 38]. Biological CFF can be actuated by different means. Unlike the vortex-based centrifugal CFF that likely predominates in gill rakers [33, 35–37], balaenid intraoral CFF involves pressure gradients.

Because previous flow tank experiments [24, 25] suggested the possibility of some degree of cross-flow filtration in the mouth of bowhead and right whales, a more in-depth kinematic study was undertaken to test this hypothesis with a range of particle flow experiments using endoscopic videorecording, basic particle image velocimetry (PIV), and pressure transducer and flow meter data. Other empirical aspects of balaenid research (oral dissection, field observation, mathematical and physical modeling) were scrutinized to provide additional lines of evidence with which to evaluate the likelihood of balaenid CFF. Given the regrettable current absence of *in vivo* telemetric data from directly within the mouths of feeding whales, these sources of data, taken together, provide the best current means with which to evaluate hypotheses concerning mysticete oral flow patterns.

Detailed fluid dynamics modeling was also devised, in a Baleen Hydraulic Circuit (BHC) model, to connect results of the physical flow tank experiments described here to hydraulics likely occurring *in vivo*, as well as to estimate hydraulic friction within the mouth, overall hydrodynamic drag of the body, and metabolic expenditures during feeding. The BHC fluid dynamic model and its scaling relations is sufficiently complex that it must be described in a separate sequel paper. A further extension of the BHC model will examine physiological and ecological consequences of balaenid CFF, particularly bioenergetic implications of CFF on drag generation, along with an assessment of caloric intake and estimates of metabolic costs required for CFF.

## Materials and Methods

### Rationale and overview

Fluid flow and filtration processes occurring in the buccal apparatus of a typical balaenid whale take place over several scales, including the entire mouth length (3–5 m AP), height (1–3m DV), and width (1–3m ML spanning the whole mouth, not just one side). Markedly smaller scales of 1–10cm are found over the spacing and chord of baleen, and still smaller scales of <1–10mm are relevant to the hydrodynamics of the boundary layer and turbulence between baleen plates.

Experimentally and theoretically studying the effects of varied scales spanning five orders of magnitude becomes impractical for many reasons, starting with existing flow-tank facilities built at human scale. Moreover, although high-level fluid simulation techniques such as Computational Fluid Dynamics (CFD) are in principle powerful enough to handle such a range, they can do so only at great costs in computer hardware and time. The approach applied here uses a flow tank study of *local* fluid dynamics of real, 20 cm baleen highlighted in the red dashed box in Fig 1, and uses these data in a fluid dynamic simulation of the *global* flows outlined in the dotted green box of Fig 1. Such a focus makes sense in the context of the whale buccal cavity’s exceptional serial symmetry along its anteroposterior axis, where pressure gradients and flows speeds are likely to vary little over length scales of ≤0.3 m. Thus anteroposterior flows along the baleen rack, as well as their splitting and entry through each intra-baleen (IB) channel, can be carried out with a high degree of realism (Fig 1).

### Experimental specimens

**Fig 4:**
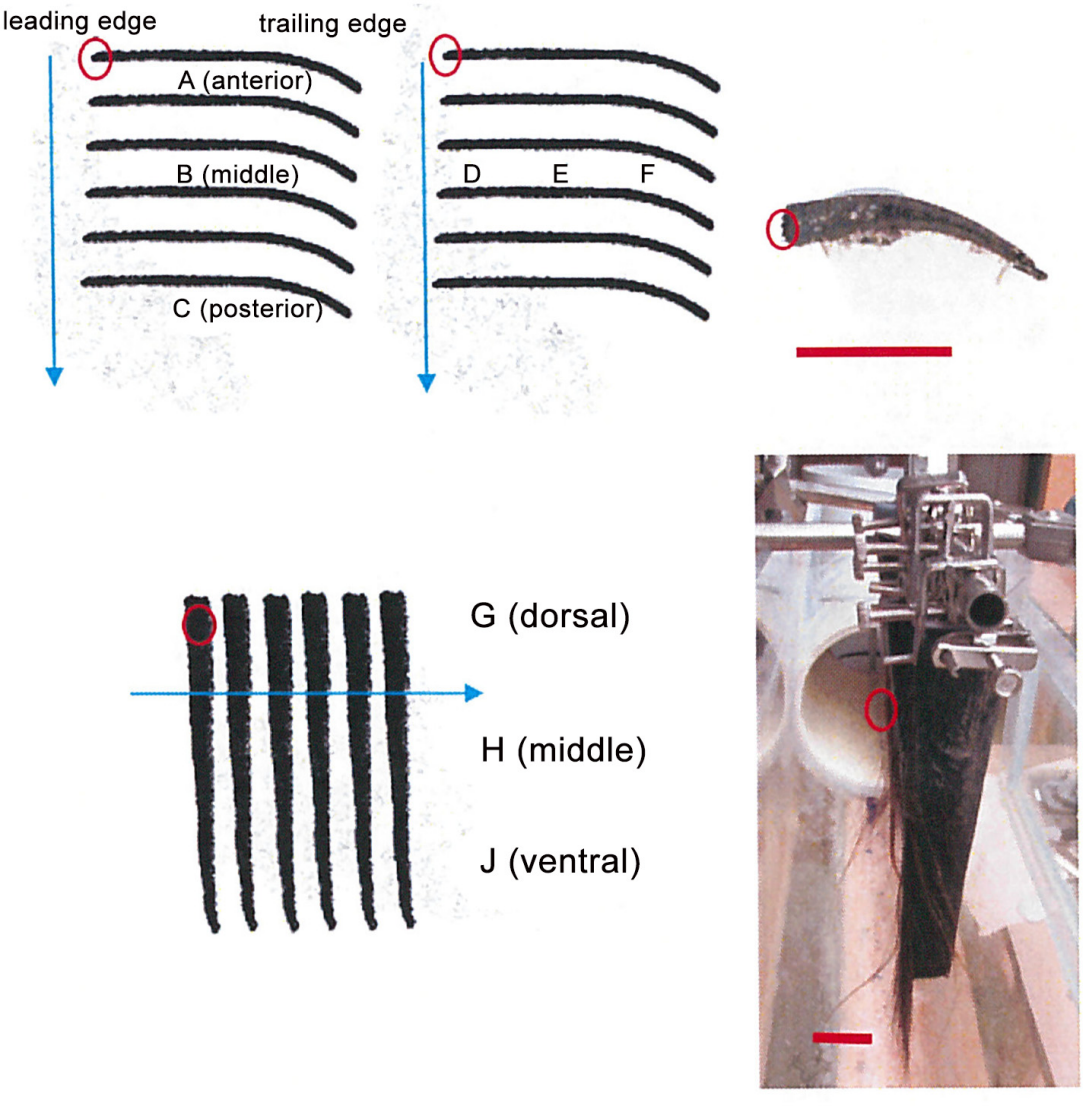
Schematic diagram showing arrangement of baleen sections into mini-racks of six plates for flow tank testing, in dorsal (top left and center) and lateral (bottom left) views. Endoscopic video sequences were shot and sensors (pressure transducers and impeller flow meters) were placed at various horizontal and vertical positions (A-J) to record flow data in multiple directions. Water flow is shown via blue arrows: in dorsal views (top left and center), from top to bottom of figure; in lateral (= medial) view (bottom left), from left to right. In dorsal views relative to whale’s oral cavity as well as flume (top left and center), sensors were placed in the same vertical position (water depth) but at varying positions anteroposteriorly, adjacent to different ‘plates’ (top left), or mediolaterally, between the same plates (top center). Lateral view also refers to whale’s oral cavity as well as flume. Clouds indicate primary disposition of flowing particles added to the flume for the experimental protocol; these accumulated mostly at the posterior and ventral regions of a mini-rack. Baleen fringes have been removed from diagrams for clarity; fringes are always located on plate’s leading edge, with position indicated by red circles. Leading/trailing edges refer to baleen location/position within whale’s oral cavity as well as within flume. Photo at top right indicates top of sectioned baleen, showing hydrofoil shape with medial lingual (leading) edge to left and lateral labial (trailing) edge to right. Photo at bottom right (same orientation as close-up photo) shows mini-rack experimental setup. Scale bars (red) = 5 cm.

Samples of bowhead baleen (20 x 7 cm rectangles not including fringe length) cut from multiple whole plates were used for flow tank testing (Fig 4). Plates were spaced 1 cm apart (= IB distance, Fig 2), as *in vivo* [21, 23–25]. Baleen samples were submerged in flowing artificial seawater for at least seven days prior to flow tank testing. All specimens were obtained from adult whales hunted by Inupiat Eskimos of Barrow, Alaska. Tissues were collected under Permit #519 issued by the National Marine Fisheries Service (NMFS) to the North Slope Borough, AK, Department of Wildlife Management. Baleen specimens are from animals of unknown sex, age, and body length. Whole plate dimensions were not recorded because some plates were damaged, but never on the fringed, lingual edge. This did not affect the smaller sections used for experimental testing. Although plate length and curvature reveal the general position within the baleen rack from which plates were taken, plates do not otherwise differ. Sections of six baleen plates were attached (spaced 1 cm apart, as *in vivo*) to create miniature racks (Fig 4).

### Flow tank testing

Baleen sections were clamped to a metal rod and submerged below the water surface in a 90 l circulating flow tank (flume). The tank is a vertical loop constructed of PVC [39]; it has a flat, transparent viewing window through which a ruled (1 cm x 1 cm) grid behind the test chamber could be seen [25]. The working section of the tank’s test chamber has a length of 70 cm and cross-sectional area of 900 cm^2^, with 4% area blockage due to the tissue samples measured with samples oriented perpendicular to the direction of flume flow as quantified by earlier research [23] (Fig 4). The rod holding the baleen specimens was secured to the top rim of the testing chamber. Flow of water through the tank was modulated three ways: selecting five motor speeds, using impellers of different diameter, and altering input voltage to the motor.

Flow velocity for the experiments varied from 5 to 140 cm s^-1^ although all results shown were collected in the range of 10 to 120 cm s^-1^. With respect to the flows entering the sub-rostral gap, such speeds accord with published swimming speeds of skimming bowhead whales [40–43]. Moreover, this range would also be consistent with the expected speed loss by the APT flows along each baleen rack due to the IB flows (Fig 2), as constrained by mass flow rate conservation (Fig 3). Along with the expectation of a near-stationary slurry near the oropharynx, using APT flow speeds as low as 5cm/s in both flow tank trials and balaenid mathematical modeling should therefore make sense. On the other hand, establishing fluid dynamic similarity, particularly in both APL and APT canals, is difficult given the lack of data on precise tongue and lip emplacement relative to baleen. Each tongue wall could be located as far as 50 cm or as close as 1.5 cm (measured from film and post-mortem examination) from baleen. It follows that Reynolds numbers based on APT gap width could be as low as R_e_ = 513 with a 1.5 cm gap open to a 5cm/s speed, or as high as 164,000 with a 20 cm gap open to a 120cm/s speed (as estimated with a kinematic viscosity of 1.46 x 10^−6^ m^2^/s). Both APT gaps have been considered here, as further explained below, to yield data that are dynamically similar to *in vivo* skim-feeding. Presuming that flow in a whale’s marine habitat is turbulent both at the surface and at depth, we also attempted to achieve a turbulence level close to real-life conditions, and specifically to avoid laminar flow within our test flume, by placing a plastic grid (of 14 x 14 mm squares) within the flume just anterior to the test chamber; this was presumed to create a realistic level of turbulence better than an initial metal screen (of 6 x 6 mm squares).

A digital impeller flow meter (Geopacks model MFP51; Hatherleigh, Devon, UK) with impeller diameter of 0.918 cm was used to measure flow velocity before, during and after experimental trials. Separate trials without baleen samples, but with two parallel plastic plates forming channels oriented parallel to the tank’s main flow (and with the impeller placed directly in between and precisely in the middle of the two plastic plates), were carried out to assess effects of effects of blockage caused by the presence of the impeller in narrow canals, in particular IB canals. The parallel plastic plates were gradually brought closer to form incrementally narrower channels. Both actual and measured flows (i.e., from the flume’s water flow speed when measured with no experimental apparatus present *versus* impeller recordings taken with the plastic or baleen plates present) remained the same within sampling errors down to channel widths of 5cm. Measurements at channel widths of 4cm, 3cm, 2cm and 1cm necessitated correcting the measured value by factors of 1.03, 1.04, 1.07 and 1.14 respectively. By readjusting the impeller position during trials with baleen, this flow meter was also used to compare intrabaleen (IB, AKA transverse or ML) flow velocities between plate sections with the linear flow velocities of bulk fluid through the test chamber, as well as with the AP flows moving immediately past selected baleen. No additional correction factors were determined to be needed when trials were performed with the impeller adjusted or moved to record water flow speeds in varying directions (e.g., AP or IB). Tests were performed in salt water at 19°C. Plates were submerged before flow commenced. All trials were conducted five times, with pairwise ANOVA testing of data from replicates. A separate section below outlines the statistical methods used to analyze results and judge significance.

**Fig 5:**
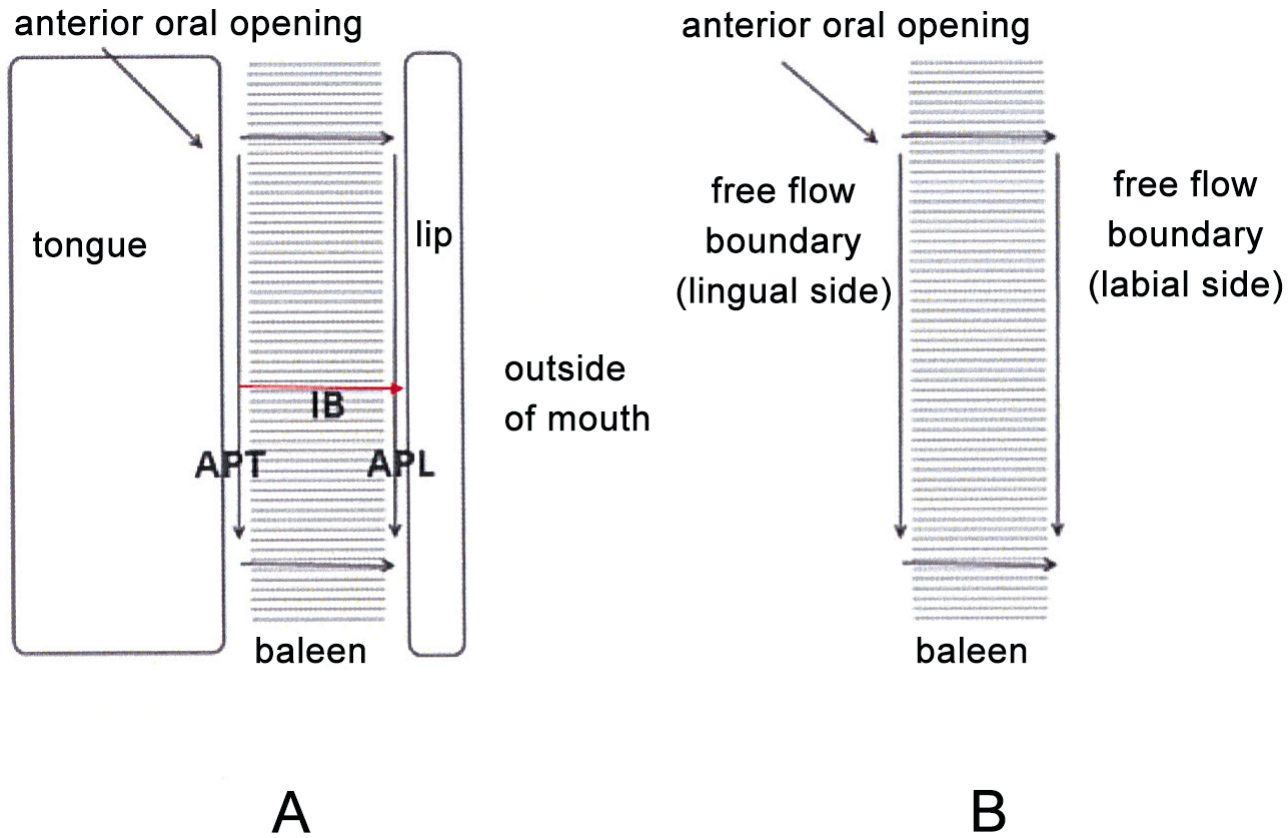
Schematic diagram indicating continuous unidirectional water flow (arrows) through idealized balaenid mouth (dorsal view), as in flow tank testing. Water enters the mouth anteriorly through an opening between paired baleen racks, flows over the tongue, and exits laterally posterior to the lip. Inside the mouth, water can flow transversely through intra-baleen (IB) channels between plates, representing IB flow (horizontal arrows) in either through-put or cross-flow filtration (TPF & CFF), or tangentially along the inside (lingual) edge of the baleen rack, representing cross-flow filtration (CFF, vertical arrows). Anteroposterior (AP) flow can be on baleen’s medial side by the tongue (APT) or lateral side by the lip (APL). Part A (left) shows initial “bounded” setup with simulated tongue and lip; later trials (B) had free boundaries. IB channels are always of the same width (1 cm).

Accurately simulating oral flow in physical and computer models is crucial; achieving proper surface orientation (i.e., angle of attack) of baleen plates and relative positions of other oral structures (e.g., tongue, lips) is essential (Fig 5). Initial “proof of concept” testing was done with louvered Venetian blinds, acting as an analogue of a baleen rack, to study the hydrodynamics of continuous water flow in the balaenid mouth. Mini-racks of these blinds, and later of actual baleen (for all results reported here), were initially joined in flow tank testing with a water-filled transparent plastic box (16 cm L x10 cm W x 12 cm H) representing the tongue and a transparent sheet of plastic (16 cm L x 1 cm W x 12 cm H) representing the lip, both of which could be filmed through to see the ruled grid (Fig 5). These two removable anatomical structures bounded the simulated intraoral flow and thus created a pair of anteroposterior (AP) channels on the medial tongue side (APT channel) and lateral lip side (APL channel), as depicted in Figs 1 and 5. It is important to note that the intra-baleen (IB) channels are always of equal width (1 cm) between adjacent plates throughout the entire baleen rack. Even as plates curve (Fig 4) adjacent plates likewise bend in a complementary manner, maintaining an equidistant IB relation as verified by measured obtained during post-mortem study of right and bowhead specimens [23, 25].

The resulting flume setups thus represent contrasting extremes of intraoral hydrodynamic flow parameters for balaenids. The first series of flow experiments involved a “constrained flow” configuration featuring simulated tongue and lip walls positioned nearest to the baleen mini-rack. The schematic plastic (Fig 5) tongue and lip were placed at a 1.5 cm (average) distance from the baleen, as suggested by necropsy data. For the second “free flow” configuration both tongue and lip walls were removed to simulate tongue and lip positions as far as 0.5 m away from baleen, as suggested by field observations of right and bowhead whale feeding.

Experiments also investigated particle capture by baleen plates and fringes, using solid, non-sticking latex polymer beads (Sargent-Welch 50024). The beads are bright blue and neutrally buoyant (1g/cc) with a mean particle size (diameter) of 710 μm (approximate diameter range = 640–725 μm), similar to the body length (prosome and metasome) of copepods and copepodites on which balaenids typically feed. Particle density was not varied but held constant at approximately 15,000 particles m^-3^, consistent with prey densities encountered by whales. Particles were added to the flume prior to experimental trials and were evenly distributed throughout the circulating flow by the action of the flume impeller. After each run of 3–5 trials all water was flushed from the flume, with new water and particles added to ensure proper particle density and circulation. Flow meter records of stream velocity were verified by video analysis of particle movement relative to the ruled background. In some instances particles made incidental contact with but did not remain trapped on baleen fringes; such particles were carried away by water flow or swept away by undulation of other fringes. For purposes of this study, particles were deemed captured if they remained in contact with baleen for at least five consecutive video frames based on the kinematic analysis of sequences described below (with video shot at standard speed, 30 frames sec^-1^). It is not known how long prey items remain attached to baleen of continuously feeding balaenids, if they do in fact become caught within the filter (less likely with CFF than TPF), but this is thought to involve seconds to minutes depending on prey density [22, 25].

Along with flow velocities, pressures were directly recorded at varying sites within the baleen mini-racks (Fig 4) using SPR-524 3.5F Millar Mikro-Tip^®^ micromanometer-tipped catheter pressure transducers (20 ms response time; Millar Instruments, Houston, TX, USA) with bare 1 mm^2^ tips oriented vertically or horizontally and catheters taped to the metal rod securing the baleen sections. Pressures were recorded at all experimental flow velocities. Transducers were linked to Power Lab units running the Chart application (AD Instruments, Colorado Springs, CO, USA) on Dell Optiplex 745 computers, and were calibrated with Millar PCU-2000 control units recording at 100 kHz sampling rate (16 bit resolution digitization).

### Kinematic analysis

Kinematic sequences were videotaped from the viewing window or underwater from the testing chamber with a digital recording endoscope (VideoFlex SD; Umarex-Laserliner, Arnsberg, Germany) with an illuminated 17 mm camera head (5/25/50 cm focal distances) that recorded JPEG still images and AVI video (standard speed, 30 frames sec^-1^). The camera was fixed in position in the testing chamber or outside the viewing window so that it could record in ambient light or using built-in illumination. Most sequences were shot from above for dorsal views and from the side for lateral view, as with pressure and flow measurements (Fig 4). Digital sequences (N = 545) were downloaded and analyzed on a Dell Optiplex 745 or Dimension D610 computer using Kinovea 0.8.15 video chronometer and motion analysis software. Each sequence lasted 20 s (total 108 min footage). Sequences were analyzed to detect flow of particles and their fate. For the kinematic analysis, the flow field was roughly rectangular in shape as defined by a distance of 4 cm from the entire “central” baleen plate within the middle of the video sequence (a span that included several adjacent plates given the 1 cm IB distance), with all visible particles analyzed during each 20 s sequence.

Following the scheme of Sanderson et al. [35], outcomes were classified as 1) no contact (particle moves through flow field without touching baleen); 2) bounce or slide (particle strikes baleen in one or more video frames but does not stop moving); 3) capture (particle comes to rest on baleen, remaining immobile for at least five consecutive video frames). Principal kinematic variables include particle velocity and acceleration, movement and spacing of free fringes of baleen, and distance along a fringe from its origin on the baleen plate, all tracked relative to observational references (fixed grid background or baleen plates), with playback at 10–100% of original speed or frame-by-frame, synchronized to time coding. The software allowed for magnification, plane perspective, tracking of path distance, and particle velocity measurement. In some trials, PIV was used to analyze reflective particle movement, with illumination using a green laser (532 nm, 1W, Nd:AG/Nd:YVO_4_) and basic lens/mirror arrangement to create a single vertical or horizontal plane of green illumination [44].

### Statistical analysis

This experimental study was set up to determine if the balaenid suspension feeding apparatus uses CFF or TPF. Accordingly, two underlying assumptions and corresponding null hypotheses are 1) that longitudinal (AP) flow through the flume’s simulated mouth and its baleen mini-rack should equal the transverse (ML) flow throughout the baleen and mouth, and 2) that flow in all directions (AP, ML, DV) will be of the same magnitude at all positions through the simulated mouth. Thus ANOVA testing was designed not as a global assessment of replicate data but with multiple pair-wise comparisons to determine if differences in flow rates at various locations were statistically significant (for example, comparing flow at position A shown in Fig 4 to flow at position B and to flow at position C). Tukey’s range test was then performed in conjunction with the ANOVA as a post-hoc test to determine if means were significantly different from each other.

However, a major concern was that these flow rates might not all be independent of each other but rather linked together (leading to lower likelihood of their being significantly different). In other words, substantially different (either lower or higher) flow at one position, such as A, might lead due to conservation of mass to a correspondingly higher or lower water flow at other positions. Close examination of the experimental design as well as the data set in consultation with a statistician indicate a strong likelihood of independence. Nonetheless, to account for the possibility of linked variabilities, a different post-hoc test, the Bonferroni correction, was performed in conjunction with the ANOVA to deal with potential problems of multiple pairwise comparisons and counteract the possibility of linked (dependent) data sets. Because of these post-hoc adjustments (applied following ANOVA, rather than to the original raw data) we report two p values used to evaluate statistical significance, along with F ratios.

## Results

### Direct flow and pressure measurement

**Fig 6:**
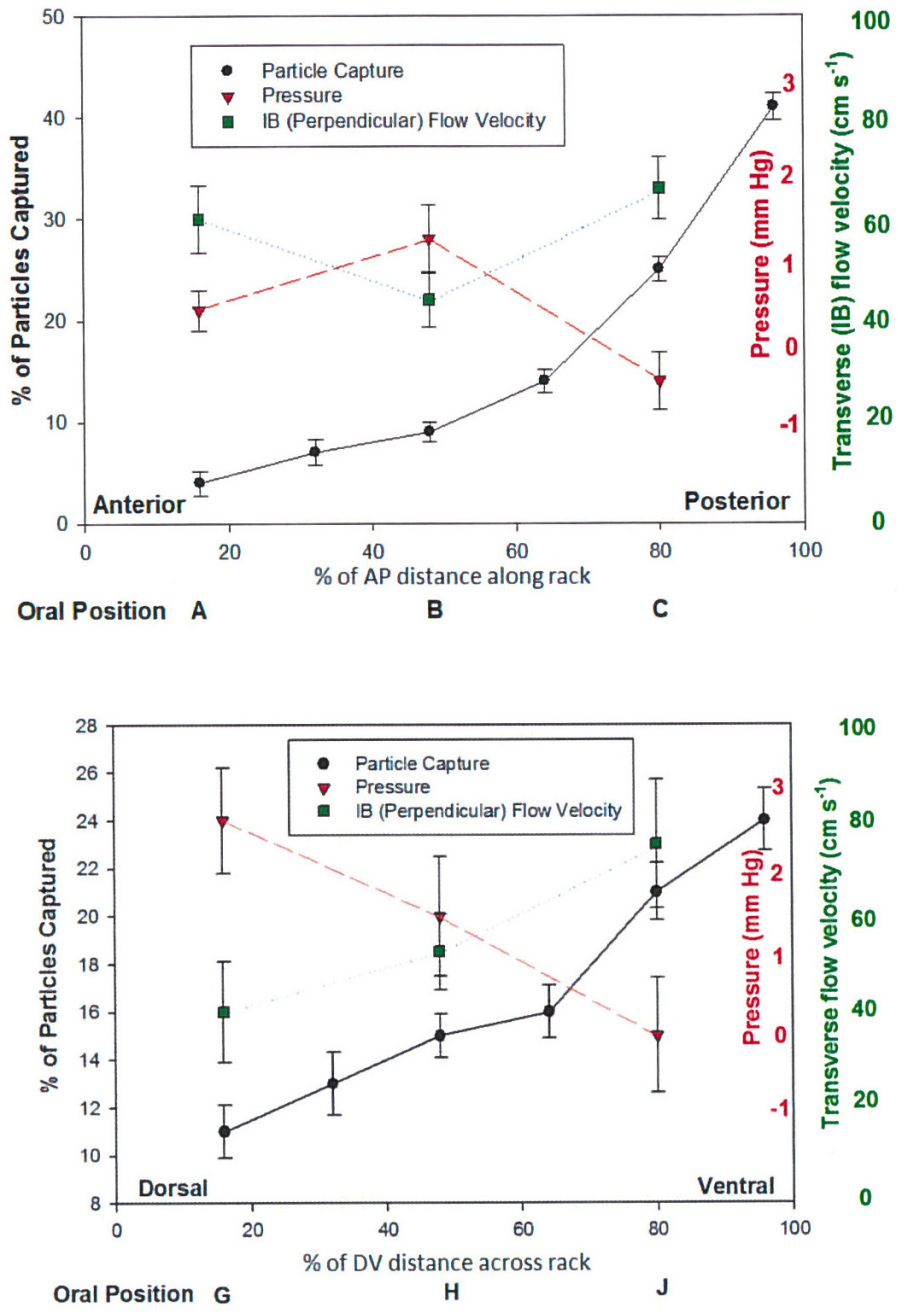
Horizontal (anteroposterior, AP, top of figure) and vertical (dorsoventral, DV, bottom) flow data through baleen sections, with sensors at three positions. For AP flow (top), many more particles were captured posteriorly rather than at the anterior of the baleen ‘rack’; flow pressure was highest at the center of the rack and lowest posteriorly; perpendicular intrabaleen (IB) flow velocity was greatest at the rear of the rack and least at its center. For DV flow (bottom), more particles were captured at the bottom of the rack than its top, with pressure declining and transverse (mediolateral) IB flow increasing from dorsal to ventral. Pressure and flow data (mean±SD) from trials with test tank flow of 94 cm s^-1^; particle capture data (mean±SD) combined from all illuminated (non PIV) trials at all flow velocities.

As water flows through a whale mouth or flow tank, it has two longitudinal) components: anteroposterior (AP) and dorsoventral (DV). Relative to a whale, these can be regarded as horizontal and vertical flow. Flow can be directed perpendicular to this longitudinal stream, turning medially (inward) toward the tongue or laterally (outward) toward the lips, and also within baleen (intrabaleen or IB flow). Initial observations with baleen mini-racks as well as preliminary “proof of concept” tests with Venetian blinds indicated that most water and particles flow posteriorly, with the current of the flow tank’s stream, along the medial edge of the plates (between simulated tongue and baleen rack; Figs 1–5) instead of turning and passing transversely between plates, perpendicular to the longitudinal flow (Fig 6). In addition to initial observations indicating AP flow (from front to back of simulated mouth), DV flow occurred through the mini-rack. Trials were conducted with and without buoyant particles not only to study the hydrodynamics of prey capture but also to test differences, if any, between “clean” and “clogged” baleen filters.

**Fig 7:**
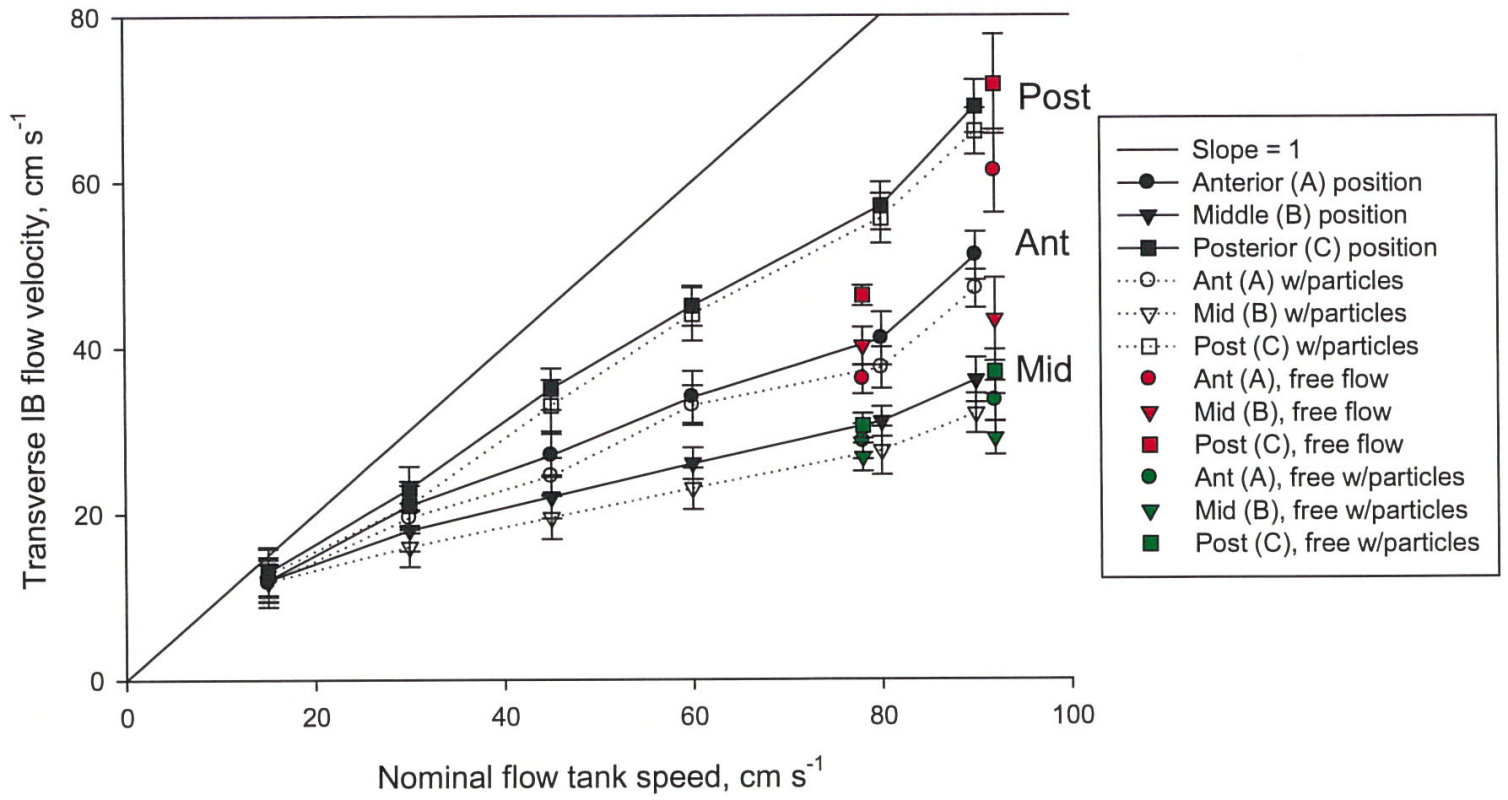
Flow velocities at three positions within baleen rack (shown in Fig 3) showing that velocity of water flowing anteroposteriorly through simulated mouth is not matched by equal perpendicular intra-baleen flow (= mediolaterally between baleen plates of the mini-rack). This plot combines data from 32 trials, including 18 (56% of all trials) bounded by simulated tongue lips and 14 (44%) in free-flow conditions. At all linear flow speeds, perpendicular IB flow (mean±SD shown) is greatest at the rack’s posterior and least in its center. With buoyant particles in water (simulating copepod prey), transverse flows (dotted lines) decreased slightly at all locations. In free flow conditions (with no simulated tongue or lips), IB flow further decreased.

A straightforward means of evaluating hypotheses of perpendicular TPF vs. tangential CFF involved testing water flow velocities through plates of the baleen filter (= intra-baleen or IB flow) compared to the flume tank’s longitudinal AP current flow. The standard TPF model suggests that water entering the mouth passes evenly between plates, so that particles (prey items) can be trapped along the entire length of the baleen rack. This would mean that at all anteroposterior points along a rack, water flows mediolaterally, from baleen’s lingual to labial side (Figs 4 and 5) as IB = AP flow as measured by flow speed (i.e., longitudinal flow through the mouth equals laterally-directed flow between adjacent baleen plates). Flow meter analysis from all tested flow speeds (5–140 cm s^-1^), including bounded and free flow conditions, reveals that IB<AP flow; perpendicular (mediolateral) flow seldom if ever matches longitudinal flow of water in the test tank (Fig 7). Moreover, IB<AP remains the same quantitatively, regardless of the presence or absence of the tongue-lip structural relation, strongly suggesting the existence of IB flows as a result of baleen geometry alone rather than of specific AP canal geometry (and dimensions). Note that mediolateral IB flow is lowest in the center of the baleen rack and greatest at the rack’s posterior (Figs 6 and 7) based on pairwise comparison. [For the center compared to anterior, N = 23, F(1, 22) = 6.34, p = 0.04, p = 0.06 with Bonferroni correction; for center compared to posterior, N = 23, F(1, 22) = 8.12, p = 0.05, p = 0.07 with Bonferroni correction.] Generally, having an increase of the IB flow posterior to the last baleen plate (position “C” in Fig 4) is an artifact of the experimental setup, where a good part of the APT flow is allowed to travel mediolaterally and not through a narrow channel (as in positions “A and “B”). Interestingly, a similar effect is expected to occur *in vivo*, as the entire APT flow passing the last baleen plate will turn laterally and onto a narrow canal formed by the oropharyngeal wall and baleen plate before heading laterally to the PO. This mediolateral flow should move at higher speed if the canal is narrower than the APT gap, again per mass flow rate conservation.

In the dorsoventral plane, mediolateral IB flow was greatest in the ventral and least in the dorsal locations (Figs 6 and 7). Likewise more particles made contact with and were captured by ventral-most baleen (Fig 7). Recorded flow speeds dropped slightly but not significantly (Fig 6) in trials with particles vs. trials without particles (N = 18, F(1, 17) = 9.25, p = 0.11, p = 0.12 with Bonferroni correction). Flow speeds also dropped in free flow vs. constrained conditions; both showed more DV than IB flow (Figs 6 and 7; N = 20, F(1, 19) = 12.83, p = 0.09, p = 0.10 with Bonferroni correction).

Microtip pressure transducer measurements at various locations in the constrained flow setup (Fig 5) reveal lower pressures at the posterior and ventral regions of the baleen mini-rack (Fig 6; for pairwise comparison of posterior to anterior, N = 24, F(1, 23) = 7.71, p = 0.11, p = 0.14 corrected; for posterior to mid, N = 24, F(1, 23) = 6.98, p = 0.07, p = 0.08 corrected; for ventral to dorsal, N = 17, F(1, 16) = 5.58, p = 0.06, p = 0.09 corrected; for ventral to mid, N = 19, F(1, 18) = 10.04, p = 0.11, p = 0.10, p = 0.12 corrected). Where the lowest pressures were recorded, flow speeds were highest, suggesting a simple hydrodynamic flow regime in both AP and DV planes.

We are unsure why transverse flow was slower with particles present (Fig 7). The expectation is for trapped particles constricting a channel such as the IB gap to accelerate flow by a simple Venturi effect. An alternate explanation could be the thick slurry of accumulated particles creating smaller canals within the IB channels, which in turn may have slowed flow via viscous friction (similar to Hagen-Poiseuille flows in narrow pipes).

### Prey capture

**Fig 8:**
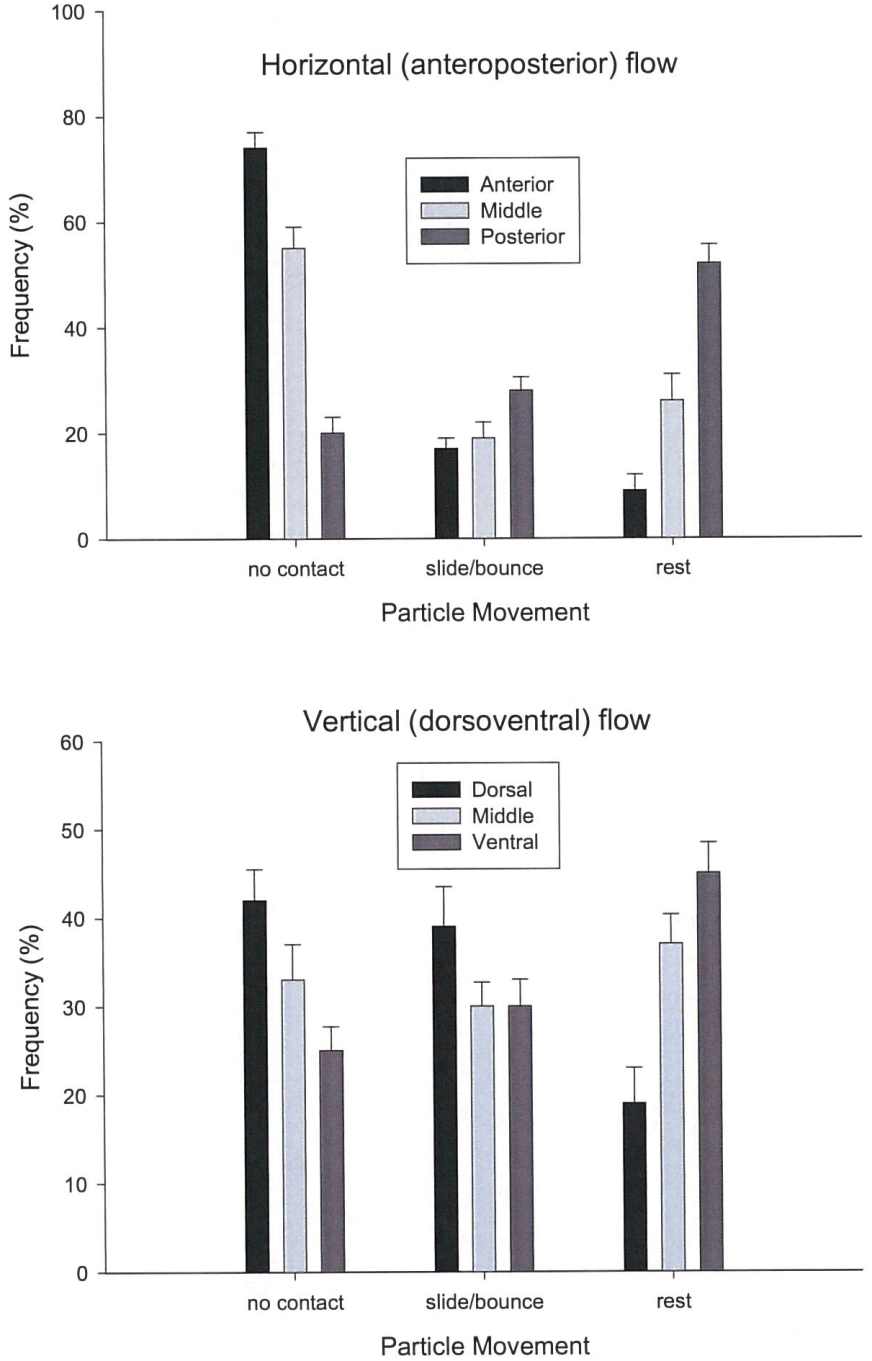
Fate of particles (mean±SD) moving along and through mini-racks in horizontal (anteroposterior, top of figure) and vertical (dorsoventral, bottom) directions. Data are from trials with flow velocity 80 cm s^-1^. Particles either moved without contacting the baleen plates/fringes, bounced or slid off baleen before proceeding posteriorly or ventrally, or were captured and came to rest, remaining immobile in contact with baleen for at least five consecutive video frames. Particles were much more likely to rest in posterior and ventral regions of the baleen racks, suggesting tangential cross-flow filtration (CFF) rather than through-put filtration (TPF) in both AP and DV directions.

Kinematic testing of the fate of particles also confirmed flow observations of experimental trials. Pairwise comparison revealed that particles were significantly more likely to make contact and be captured by baleen fringes at the mini-rack’s posterior plates (Fig 8) rather than middle (N = 25, F(1, 24) = 8.83, p = 0.02, p = 0.04 corrected) and anterior locations (N = 25, F(1, 24) = 14.33, p = 0.04, p = 0.05 corrected). Analysis of endoscopic videotaped sequences indicates that particles mostly made no contact with baleen fringes near the anterior and middle of the mini-rack. This finding held true for all tested flow speeds. No differences in particle movement were found in sequences recorded under normal illumination relative to sequences laser-lit for PIV analysis, except that not all baleen fringes could be clearly seen in PIV. Otherwise, the speed and direction of particle movement was the same in normal and laser-illuminated video sequences.

Particles in AP flow along the baleen mini-rack’s medial (lingual) margin also tended to move, and be preferentially captured, ventrally (Fig 8). Particles were significantly less likely to make contact on the dorsal region of baleen plates (relative to mid, N = 14, F(1, 13) = 5.89, p = 0.03, p = 0.04 corrected; relative to ventral, N = 14, F(1, 13) = 7.84, p = 0.04, p = 0.05 corrected) and were more likely to make contact with ventral fringes at all flow speeds. When data from all trials (all flow speeds) were combined, particle accumulation in the posterior and ventral regions of the simulated balaenid mouth becomes even more apparent (Fig 6).

To show that these findings were not artifacts of the rack’s positioning within the flume’s water column, all components of the simulated balaenid filtration apparatus (baleen mini-rack, tongue and lip) were suspended in the tank at varying depths, from the surface to the bottom. In all cases the same findings were obtained: significantly more particles were captured by (i.e., came to rest on) baleen fringes in the posterior and ventral regions (for posterior compared to anterior, N = 025, F(1, 24) = 9.39, p = 0.03, p = 0.06 corrected; for posterior compared to mid, N = 25, F(1, 24) = 8.40, p = 0.05, p = 0.06 corrected; for ventral compared to dorsal, N = 22, F(1, 21) = 10.09, p = 0.04, p = 0.05 corrected; for ventral compared to mid, N = 22, F(1, 21) = 6.77, p = 0.02, p = 0.03 corrected). For AP flow, particles also bounced or slid (made momentary contact) more in the mini-rack’s posterior than anterior or middle regions; in the dorsoventral plane, in contrast, particles bounced or slid off baleen more in the dorsal region than where they were predominantly captured (i.e., came to “rest”) in the ventral area (position J of Fig 4, as shown by capture data presented in Fig 8).

Regarding transverse IB flow (Fig 5), particles were found to be captured mainly on the medial (lingual) side or “leading edge”, where baleen is frayed into a mat of long fringes. Although almost all contact occurred on baleen’s medial side, some particles that were not “captured” continued to flow laterally, so that there was flow of particles, and bouncing and sliding, at all locations. Particles that reached the most lateral (labial) position on a plate’s trailing edge had always made some contact, usually by sliding along or bouncing off the middle region before exiting laterally.

## Discussion

The finding that particles were much more likely to contact baleen in posterior and ventral portions of the shortened racks strongly suggests two degrees of directionality to the flow field within the simulated whale mouth. Specifically, a substantial element of tangential rather than through-put IB flow in AP and DV directions (Figs 6–8) is apparent. These are wholly indicative of CFF instead of the traditional view of TPF within the balaenid mouth. Particle capture data are further supported by flow and pressure measurements, all of which indicate that intraoral flow is predominantly AP and DV rather than mediolateral (IB) as the standard TPF model predicts, where all flows advance in the same direction.

The extent to which tangential flow of prey-laden water along the fringed lingual surface of the baleen rack creates a pressure differential that aids or inhibits mediolateral flow through the filter’s anterior and middle regions is unknown, although this may be possible [38]. This study strongly suggests the existence of mediolateral IB flows—and therefore CFF—as the result of baleen geometry alone, particularly with regard to their curved trailing edge (Figs 4 and 7). From this feature and the inherent narrowness of the IB canals, as depicted in Fig 2, APT flows lose speed due to fluid mass loss through the baleen per mass flow rate conservation (Fig 3). The entire process is primarily driven by the ram flow into the mouth and maintained by the existence of lower external pressures near the PO [23–25]. Thus an overall pressure gradient forms anteroposteriorly. Another mediolateral pressure gradient is created by the baleen geometry that generates the IB flows and modulates the AP pressure gradient on the labial side of the mouth. Depending on shear forces, a small layer of accumulating retentate can still form on this surface due to concentration polarization or other boundary effects [45–48]. The scale of industrial CFF is much smaller than that of whale feeding, with molecular and adhesive processes governing commercial applications instead of high Reynolds numbers expected to apply in the whale mouth [25]. The large-scale flow of mysticete oral filtration likely would not involve the concentration polarization and other effects described in industrial circumstances, where very small (0.01–1 μm) particles are typically filtered in micro- and ultrafiltration [30–32, 38, 49–51].

Actual flow speeds are controlled by muscular actions of the tongue and lips in modulating APT and APL channel width, as determined by this study and previous work [23–25]. Findings from this study suggest that the lips rotate laterally to open the APL canal to a uniform width, generating flow separation which with accelerating external flows creates a region of low pressure leading to minor suction within the buccal cavity, thereby initiating CFF. As more copepods become trapped in the fringe mat [24], APT flows slow, increasing overall intraoral hydrodynamic friction [23].

Our results bring further insight on the role of overall body size in effecting CFF, at least in balaenids. From the general principle of flow mass rate conservation within pipe networks (Fig 3) it is clear that adding more outlets, filter pores, or baleen canals, or in other words adding more filter surface area, is beneficial as this leads to slower through-filter (IB) flow speeds, and therefore longer times until the filter becomes clogged. This follows from any fluid entering an inlet (*A*_*in*_) and losing mass through multiple (*N*_*out*_) outlets of surface area *A*_*out*_ as follows (Fig 3): *ρU*_*in*_*A*_*in*_ *~ ρA*_*out*_ *N*_*out*_*‹U*_*out*_*›* with *U*_*in*_ and *‹U*_*out*_*›* being the flow speed through the inlet and flow speed averaged over all of the outlets, respectively. With the product *A*_*out*_ *N*_*out*_ corresponding to overall filtering surface area, adding baleen canals (filter surface area) decreases outlet flow speeds by a factor *A*_*in*_/*A*_*out*_*N*_*out*_. Thus larger CFF surface area further aids in lengthening clogging time by maintaining fast tangential flows and transporting captured prey directly to the oropharynx without using the tongue as a broom to remove prey from the filter [22]. On the other hand, having an IB flow fastest at the posterior end of the rack is also supported by our computer modeling (JP unpublished data), as a result of a much reduced lingual AP flow re-joining, behind the last baleen plate, with a much faster labial AP flow. From our physical flow tank experiments the agreement is qualitative rather than quantitative, as the baleen rack studied in the flume is much shorter than the rack of an actual whale specimen (6 six baleen plates versus 300 or more).

Given the 1 cm IB gap, a sure way to increase filter surface area is by increasing the number of baleen plates in each rack. At fixed AP output to input flow speed, mass rate conservation (Fig 3) suggests that the average ‹*U*_*IB*_› will scale roughly with the inverse of baleen plate number. Thus increasing the plate count from 50 to 150 to 300 per rack decreases the flow splitting by factors of 3x and 6x, respectively, relative to a plate count of 50.

Other aspects of balaenid research provide multiple lines of independent evidence that strongly support the CFF hypothesis in Balaenidae. In previously unpublished limited field reports (with videorecordings and still photography, anecdotal accounts and observations) of foraging balaenid whales (North Atlantic right whales in the Gulf of Maine and bowhead whales in the Chukchi and Beaufort Seas) and the flow fields around them, water is seen exiting the mouth behind the lip, yet with little apparent flow along the medial (inner) margin of the lip along the orolabial sulcus, as would be expected if there were substantial mediolateral IB flow as in the TPF model. Clearly, the best evidence would come from *in vivo* telemetry studies using intraoral recorders swallowed or fixed inside the mouth. Flow tank and computer modeling are a lesser alternative than biologging but the best available option at present. Although direct investigation of flow within the mouth of an actual whale would obviously be desirable, physical flow tank trials and computer models nonetheless allow for experimental simulation under a wide range of flow parameters, thus providing useful knowledge that would not be possible by *in vivo* study alone.

CFF was observed in both “constrained” and “free flow” setups and with similar AP and IB flow rates (Fig 7). These data thus suggest that the presence of baleen alone is sufficient to generate the pressure gradients necessary to split the incurrent flow entering the subrostral gap. It is important to note that baleen plates are not simply flat plates aligned perpendicularly to the longitudinal AP flow within the mouth. Rather, plates are cambered on the “trailing” (lateral = labial) edge, with a hydrofoil shape (Fig 4). In contrast, the “leading” edge (on the medial = lingual side) is straight (Fig 4). Because each plate displays this curvature, flow along the lateral edge of each baleen rack (through the APL channel) differs from flow through the central APT channel in the mouth’s midline, over the tongue.

Initial proof-of-concept trials conducted with Venetian blinds aided in determining the experimental setup, but it is important to recognize that individual baleen plates and full racks involve many curvatures that play a role in altering flow. Preliminary results of flow tank experiments with pressure transducers and load cells suggest that plates may generate lift-like forces to reduce drag (parasitic form and friction drag encountered by the baleen filter from oncoming flow), which is unsurprising considering their hydrofoil-like cross-sectional shape (Fig 4). These results were obtained at flow speeds (0.5–1 m s^-1^) experienced by foraging balaenids. At the very least, we believe complexly cambered baleen plates and fully curved racks serve as vanes to direct water flow, potentially reducing drag and improving filtration and likely facilitating CFF over TPF. In functional terms, the consequence of the baleen curvature is that these effects cause a pressure differential chord length-wise. Further study is needed to resolve these questions; experiments could compare normally curved baleen with flat lamellae. We are investigating, through our detailed fluid dynamic model, the physics of this flow stream, particularly the role of balaenid morphology in creating and sustaining the pressure differential that generates IB cross flow currents.

Preliminary anatomical and morphometric data from bowhead whales harvested by Inupiat subsistence hunters in Barrow, Alaska, such as wear patterns on baleen and along the orolabial sulcus, are largely conjectural yet also support the CFF hypothesis. More compelling are previously unpublished findings from dead balaenid whales, hunted and stranded. Consistently, baleen plates and fringes are entirely or nearly devoid of prey items or ectoparasites. The few copepods or other prey that are (very rarely) found within the baleen filter are typically located in posterior portions of baleen racks [22].

It has been presumed that as with any continuous dead-end sieving, particles (retentate) separated from flushed water would accumulate and obstruct or clog the balaenid oral filter. To relieve the filter of this cake formation and maintain functionality the filter would require periodic cleaning. Werth [22] posited three possibilities for cleaning baleen: 1) direct dislodging via mechanical scraping (e.g., tongue elevation and retraction); 2) indirect mechanical removal via head shaking or nodding; 3) hydrodynamic backwash flushing by reversing flow through baleen (most likely via rapid lingual depression). Not only would CFF largely preclude filter clogging and hence eliminate the need for cleaning, but it would also explain why baleen is not found with entangled prey. This is especially remarkable considering the large antennae and other bristly appendages of balaenids’ preferred planktonic prey (predominantly copepods), which might easily snag on the dense meshwork of very fine, hair-like baleen fringes.

Results of this investigation suggest long-standing, traditional views of balaenid feeding must be revised or at least reconsidered. Recent experiments [25] have shown that baleen has variable, flow-dependent porosity, which allows it to perform its vital function in a highly dynamic environment. The conventional wisdom is of 1) static baleen, 2) sieving (dead-end TPF), and 3) a periodic need to clean a clogged oral filter. The new experimental evidence presented here indicates: 1) dynamic baleen with variable porosity; 2) predominant CFF instead of through-put sieving; 3) minimal clogging and thus little or no need to clean the oral filter; 4) prey accumulation in a dense slurry near the oropharynx, where it can readily be swallowed; and 5) some degree of DV along with primarily AP oral flow.

The relative importance of mediolateral (transverse) IB flow has yet to be resolved. Prey buoyancy will also have major consequences for mysticete filtration [22, 24, 38], as will prey density [24, 52, 53]. Further flow tank studies and modeling can further clarify the role of body size scaling, as well as of the IB gap between plates. Do narrower gaps improve or impede IB flow? Clearly, more work is needed, including large-scale flow studies with full baleen racks, additional biomechanics and materials testing, and further CFD analysis. It is hoped that *in vivo* data can be obtained from within the mouths of feeding whales to accurately visualize and quantify flow fields within the mouth. Ontogenetic changes in form and function that occur during weaning with the shift from suckling to adult feeding deserve special attention, as do the changes that occur due to the growth of baleen throughout a mysticete’s life history. It is possible that mysticetes facultatively alter intraoral flow regimes during foraging bouts, perhaps by lingual and lip movements, to increase prey capture rates [23, 54].

If CFF rather than TPF predominates in balaenid feeding, as our findings strongly indicate, standard descriptions of baleen acting as a “sieve” are at best incomplete and at worst wholly inaccurate. Although mysticete feeding qualifies as filter feeding, it may be better characterized as suspension feeding [27, 28] given that simple sieving or other direct particle impaction or interception [26] appear not to be involved, at least in balaenids. Although this investigation focused solely on bowhead and right whales, preliminary tests of baleen [25] from the humpback whale (*Megaptera novaeangliae*) suggests CFF plausibility in other mysticetes (rorqual and gray whales) that feed via intermittent filtration. Patterns of flow during expulsion of a single mouthful of water are not well known, and depend on inertial forces from gape closure, eversion of the ventral pouch and tongue, and elastic rebound [8, 55]. In at least some cases, water flow of intermittent filtration may involve gravity due to directional foraging behaviors (vertical lunges, lateral rolls) or may involve other forces (e.g., intraoral benthic suction in gray whales).

Continuous flow in fluids typically leads to formation of a vortex or multiple vortices [56]. Indeed, Vogel [39] goes so far as to assert that for steady flow in bounded systems, “truly noncircular motion must be a special case.” Vortices readily form during sustained flow in a pipe-like system [57], which characterizes the balaenid mouth [23]. Intriguingly, Pinto [38] suggested that intraoral flow may involve vortices or centripetal cyclones in rorquals (Balaenopteridae), whose intermittent ram-driven filtration involves discrete, explosive lunge-feeding events. Such vortices may play a major role in CFF by sustaining strong flow patterns tangential to, rather than perpendicularly through, baleen racks. The possible role of cyclonic vortices in both steady-state and intermittent intraoral filtration in mysticetes warrants further investigation (ongoing) with flow experiments and computer modeling. Future work also involves examination of macro- and microscopic wear patterns or scouring of baleen.

The extraordinary length (3–4 m) of baleen in adult bowhead and right whales relative to baleen in rorqual and gray whales (averaging 60 cm and 40 cm in adults, respectively [22]) may merely provide the continuously feeding balaenids, which filter tiny prey (1 mm copepods to 1 cm amphipods), with a greater surface area for filtration, especially when considering that their baleen plates (laminae) are narrower than in other mysticetes. However, it is possible that the unusual length of balaenid baleen relates to the dorsoventral aspect of intraoral flow that was revealed by this study, and perhaps also to the flow vortices suggested here, both of which may contribute substantially to generating and sustaining CFF flow patterns.

A dedicated focus on the mechanisms of mysticete filtration will yield numerous applications, including knowledge of mysticete functional morphology, comparative physiology and energetics (particularly of the drag engendered by filtration), anatomy and biomechanics, and basic foraging ecology (prey size and type, etc.). There are major implications for mysticete conservation and population management, especially related to the consequences of oral entanglement [58] or baleen fouling and substance (e.g., petrochemical) accumulation/ingestion during foraging [21, 59, 60]. We will address these concerns in a sequel paper using computer modeling to extend and apply the experimental data presented here.
